# The Clinical Significance and Prognostic Value of HER2 Expression in Bladder Cancer: A Meta-Analysis and a Bioinformatic Analysis

**DOI:** 10.3389/fonc.2021.653491

**Published:** 2021-09-01

**Authors:** Kai Gan, Yue Gao, Kuangzheng Liu, Bin Xu, Weijun Qin

**Affiliations:** ^1^Department of Urology, Xijing Hospital, Fourth Military Medical University, Xi’an, China; ^2^Surgical Research Center, Institute of Urology, Medical School of Southeast University, Nanjing, China

**Keywords:** bladder cancer, HER2, clinical significance, prognostic value, meta-analysis

## Abstract

**Objective:**

Human Epidermal Growth Factor Receptor 2 (HER2) is highly expressed in multiple malignancies and associated with patients’ prognosis, but its role in bladder cancer (BCa) remains elusive. We conducted this meta-analysis to explore the clinical significance and prognostic value of HER2 in BCa.

**Methods:**

PubMed was searched for studies published between January 1, 2000 and January 1, 2020. The odds ratios (ORs) and hazard ratios (HRs) with 95% confidence intervals (95%CIs) were used to investigate the relationship between HER2 and BCa pathological features. TCGA was mined for the information regarding as well.

**Results:**

Our study included 14 articles enrolling 1398 people. Expression of HER2 is higher in bladder cancer than in normal tissues. HER2 over-expression is associated with CIS, multifocal tumor, large tumor size, high tumor stage and grade, lymph node metastasis, progression, recurrence and papillary tumor. We could not find a significant association between HER2 expression and survival time in BCa patients.

**Conclusions:**

Our meta and bioinformatic analysis indicated that HER2 expression was related to pathological malignancy and poor prognosis in BCa.

## Introduction

Bladder cancer is the ninth most common malignancy worldwide (1), accounting for 4% of all cancer deaths among the male population in the United States in 2019 (2). According to the latest statistics published recently, 83,730 estimated new cases and 17,200 related deaths were expected in the United States in 2021 (3).

Currently, the treatment outcomes associated with bladder cancer are not satisfactory. Even after complete transurethral resection of bladder tumors or even secondary surgery, 33% of these patients will develop muscle-invasive bladder cancer (MIBC) (4). In previous studies, some urinary bladder cancer biomarkers, such as NUMA1 and CFHR1, have been proposed. The study of molecular markers in bladder cancer is gradually becoming hot (5). However, specific pathological markers clearly associated with bladder cancer are still lacking. We need better relevant markers to assess the risk of bladder cancer and predict cancer development.

The human epidermal growth factor receptor-2 (HER2) is known to contribute to cell growth, survival, and migration as a member of the transmembrane receptors (6). In addition, HER2 has been extensively studied as a tumor therapeutic target and is considered to act as a very important prognostic and therapeutic marker for breast cancer. The expression of HER2 molecules in breast cancer can already be precisely detected (7, 8). Overexpression of the HER2 gene is highly correlated with malignancy and poor prognosis of breast cancer. HER2 can be involved in the signaling pathway of breast cancer cells, increasing their proliferative capacity (7). HER2 has also been studied in esophageal and gastric cancers. In metastatic esophagogastric cancer with HER2 protein overexpression, pabolizumab can be efficiently combined with trastuzumab as well as chemotherapy. This combination therapy can benefit patients more than previous monotherapies (9, 10). Referring to the results of the relevant discussions on pathology at the 2nd International Consultation of Bladder Cancer, HER2 overexpression was associated with high-grade uroepithelial carcinoma and muscle invasion (11). A number of other similar studies have been conducted in the past and reached similar conclusions.

A previous study has reported that HER2 expression was associated with poor prognosis in BCa (12). In another study, the authors claimed that HER2 could be used as a diagnostic marker in bladder cancer especially NMIBC (13). Thus, HER2 could be considered as a useful biomarker for clinical prediction in BCa. However, the specific role of HER2 in bladder cancer needs to be demonstrated by strong evidence-based medical studies. In recent years, studies on HER2 in bladder cancer have continued to emerge in numerous journals. Thus, we performed a meta-analysis and bioinformatic analysis to assess the prognostic value of HER2 gene and protein expression in BCa patients.

## Materials and Methods

### Search Strategy

This meta-analysis was performed following the convention of PRISMA (Preferred Reporting Items for Systematic Reviews and Meta-Analysis) guidelines. An electronic search of databases from PubMed from January 2000 to January 2020 was conducted. According to the PICO framework (population, intervention, comparison, results), we used specific terms including “HER2”, “bladder cancer”, “prognosis”, “clinical significance” to search target literature. The search was restricted to English-language articles only. Two authors independently screened the title and abstract of each article and reviewed the full text. The eligibility of each article was evaluated. If there was a disagreement, the third author would join the discussion and decide whether we should include that literature.

### Inclusion Criteria

Full texts of these studies were read carefully to determine whether the articles met the following inclusion criteria: (1) The study must focus on patients diagnosed with bladder cancer; (2) HER2 protein expression was detected by immunohistochemistry (IHC). The expression level of HER2 must be clear. Referring to the “ASCO/CAP Guidelines Consensus on Breast Cancer HER2 Detection” which was jointly issued by the American Society of Clinical Oncology (ASCO) and the American College of Pathology (CAP) on December 11, 2006, if more than 10% of tumor cells show membrane staining, we call it “positive”. Otherwise, we call it “negative”. (3) Containing patient information, tumor classification, staging, and prognosis-related conditions.

### Exclusion Criteria

The exclusion criteria were set as follows: (1) The study was a review article, case report, letter, comment or conference abstract. (2) Patients were included in another study.

### Data Extraction

The extracted data for each study included the first author’s name, publication year, the proportion of cells with stained cell membrane after IHC, number of including cases, the median or mean age of patients, the percent of male patients, the median or mean follow-up, type of bladder cancer, outcome (**Table 1**).

**Table 1:** Extracted data of included studies.

Reference	Method	Number	Age	Male%	Follow-up (months)	Tumor type	Outcomes
Ding et al. (14)	IHC>10%	238	68 (Median)	81.9	47 (median)	non-muscle-invasive bladder cancer (NMIBC)	Tumor size; recurrence; progression; grade
Kolla et al. (15)	IHC>10%	90	58 (mean)	95.6	46 (median)	muscle invasive urinary bladder cancer	grade; lymph node metastasis
Krüger et al. (16)	IHC>10%	138	64 (Median)	80.4	53 (mean)	muscle-invasive bladder carcinoma	grade; lymph node metastasis
Lim et al. (17)	IHC≥50%	141	68.9 (mean)	86.5	73.3 (mean)	non-muscle-invasive bladder cancer	progression; recurrence; tumor size; grade
Hegazy et al. (18)	IHC>20%	88	not specific	not specific	36 (median)	non-muscle invasive (NMI) bladder cancer	tumor recurrence and progression; grade
Paul et al. (19)	IHC>30%	178	71 (mean)	not specific	82 (mean)	non-muscle-invasive bladder cancer	Recurrence; progression
El et al. (20)	IHC>30%	103	63 (Median)	93.2	not specific	non-muscle-invasive and muscle-invasive bladder cancer	Tumor size; Tumor grade
Inoue et al. (21)	IHC>10%	95	not specific	72.6	36 (median)	muscle-invasive bladder carcinoma	recurrence condition
Behnsawy et al. (22)	IHC>10%	161	not specific	85.1	not specific	non-muscle-invasive bladder cancer	recurrence condition
Olsson et al. (23)	IHC>30%	201	73 (Median)	83	58 (median)	non-muscle-invasive bladder cancer	tumor size, multiplicity, possible presence of histologically proven recurrence and progression; grade
Moustakas et al. (24)	IHC>10%	48	68 (mean)	97.3	not specific	non-muscle-invasive urothelial cell carcinoma of the bladder	Grade at diagnosis; recurrence-free survival (RFS)
Soria et al. (25)	IHC>30%	354	66.3 (Median)	81	123 (mean)	muscle invasive and very high-risk non–muscle invasive bladder cancer	oncological outcomes; grade
Bolenz et al. (26)	IHC>10%	198	66.7 (Median)	78.8	48.7 (mean)	non-muscle-invasive and muscle-invasive bladder cancer	recurrence; lymph node metastasis; grade
Abdelrahman et al. (27)	IHC>30%	60	52 (Median)	71.70	44(mean)	Non-muscle-invasive bladder cancer (NMIBC)	tumor recurrence, progression, recurrence-free survival (RFS) and progression-free survival (PFS); grade

IHC, immunohistochemistry.

### Study Quality Assessment

In the quality evaluation, we used the Newcastle - Ottawa Quality Assessment Scale (NOS) to evaluate the quality of the included literature. The NOS scale covers three key areas, including selection, comparability, and exposure/results. Studies with a score of seven or above are considered to be high quality on this rating scale. The results indicated that the quality of literatures we selected was not low (**Table 2**).

**Table 2:** Newcastle - Ottawa Quality Assessment Scale of the included literature.

	Representativeness of the exposed cohort	Selection of the non-exposed cohort	Ascertainment of exposure	Demonstration that outcome of interest was not present at start of study	Comparability of cohorts on the basis of the design or analysis (study adjusts for age*, sex*)	Assessment of outcome	Was follow-up long enough for outcomes to occur	Adequacy of follow up of cohorts	Total
Abdelrahman et al. (27)	*	*	*	*	**	*	*	—	8
Bolenz et al. (26)	*	*	*	*	**	*	*	—	8
Soria et al. (25)	*	*	*	*	**	*	*	*	9
Moustakas et al. (24)	—	*	*	*	**	*	—	*	7
Olsson et al. (23)	*	*	*	*	**	*	*	—	8
Behnsawy et al. (22)	*	*	*	*	**	*	—	*	8
Inoue et al. (21)	—	*	*	*	**	*	*	*	8
El et al. (20)	*	*	*	*	**	*	*	*	9
Paul et al. (19)	*	*	*	*	**	*	*	*	9
Hegazy et al. (18)	—	*	*	*	**	*	*	—	7
Lim et al. (17)	*	*	*	*	**	*	*	*	9
Krüger et al. (16)	*	*	*	*	**	*	*	—	8
Kolla et al. (15)	*	*	*	*	**	*	*	*	9
Ding et al. (14)	*	*	*	*	**	*	*	*	9

*The result of this item is positive. **There are two positive results. —The result of this item is negative.

### Usage of TCGA Database

TCGA database, a publicly available platform, was applied to investigate HER2 gene expression levels in BCa. We used R (version 3.6.3) software to Processing these data. Wilcoxon rank sum test was used to compare the expression differences of HER2 in various cases of classification. Log-rank test was used to analyze the survival database. P<0.05 was regarded as statistically significant.

### Statistical Analysis

We used the Stata 15.0 software for data analysis. For dichotomous variables including gender and percentage of carcinoma *in situ* (CIS), multifocal tumors, tumor size, stage, grade, lymph node metastases, and lymph vascular invasion, the odds ratio (OR) and 95% confidence interval (CI) were adopted. For comparison of time-related prognostic information which contained recurrence, progression, and recurrence-free survival (RFS), hazard ratio (HR) and 95% CI were applied (28). All statistical tests were two-sided, and a *P* value <0.05 was considered statistical significance. Forest plots showed the results of research analysis and publication bias was visually evaluated using funnel plot. The heterogeneity was assessed by I² statistics, and funnel plots were used to test publication bias. As noted by the Cochrane Handbook (29), when heterogeneity was less than 40%, a fixed effect model was recommended for Meta-analysis. Otherwise, a random effect model should be used.

## Results

### Search Results

**Figure 1:**
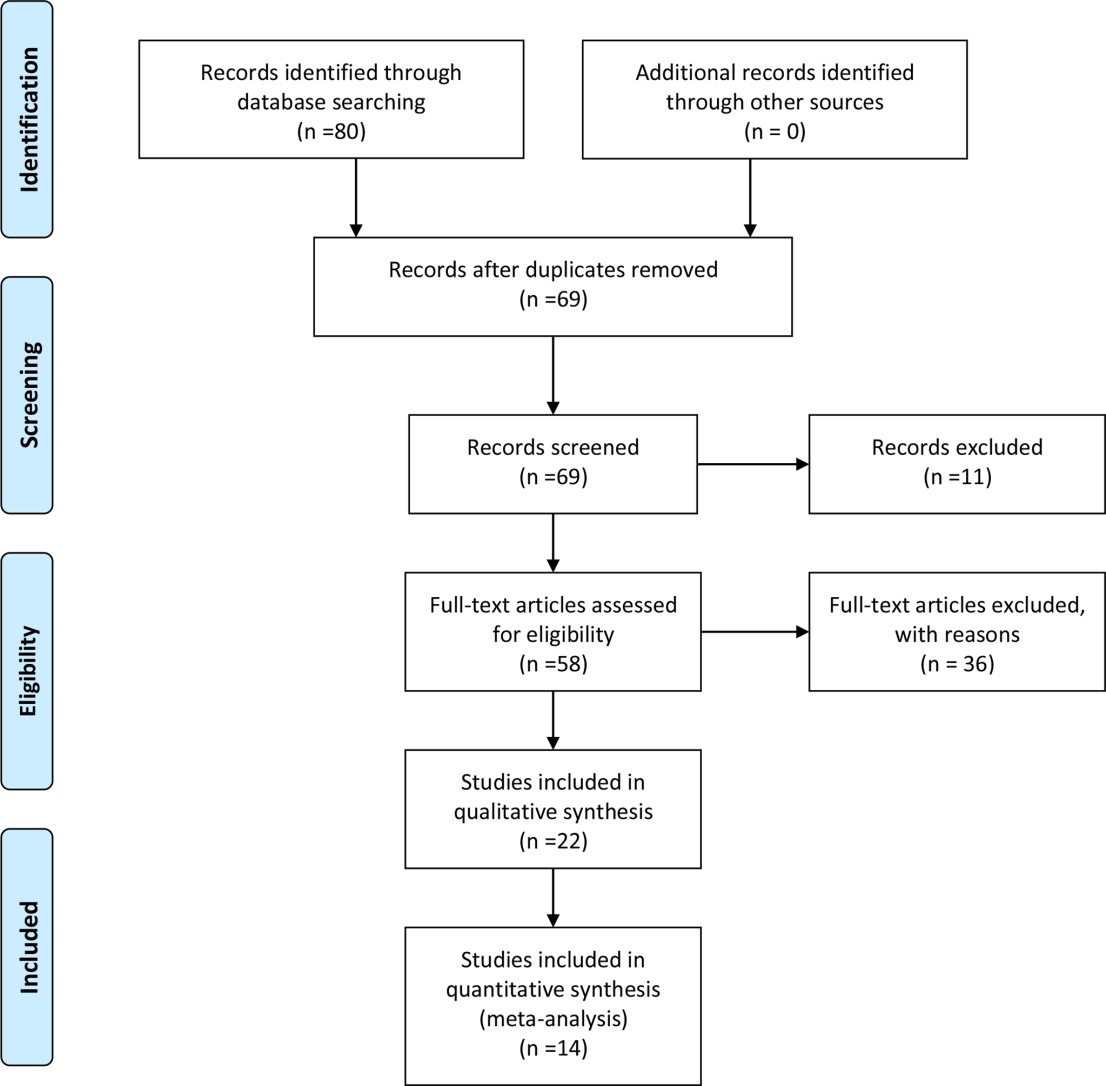
Study selection flowchart.

The search strategy retrieved 80 unique citations, of which 58 were excluded after the first screening based on abstracts and titles, leaving 22 for full article review. Finally, we included 14 articles, 1398 patients for our study. A study selection flowchart is presented in **Figure 1**.

### Comparison of Gender Differences Among Patients

**Figure 2:**
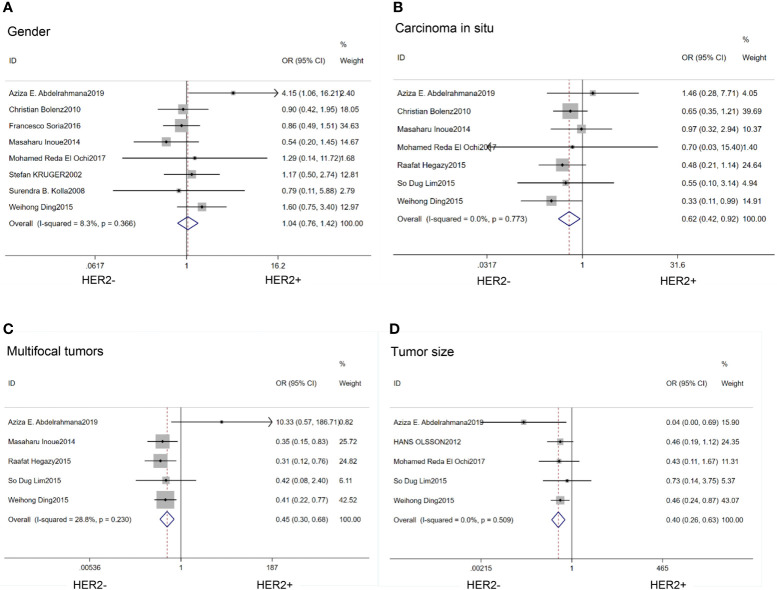
Forest plots describing the correction of HER2 protein expression with gender **(A)**, carcinoma *in situ* **(B)**, multifocal tumors **(C)** and large tumor size **(D)**. OR, odds ratio; CI, confidence interval.

There was no association between HER2 protein expression and the gender of the patients (OR=1.04; 95% CI:0.76–1.42; p=0.80) (14–16, 20, 21, 25–27) (**Figure 2A**).

### Oncology-Related Features

**Figure 3:**
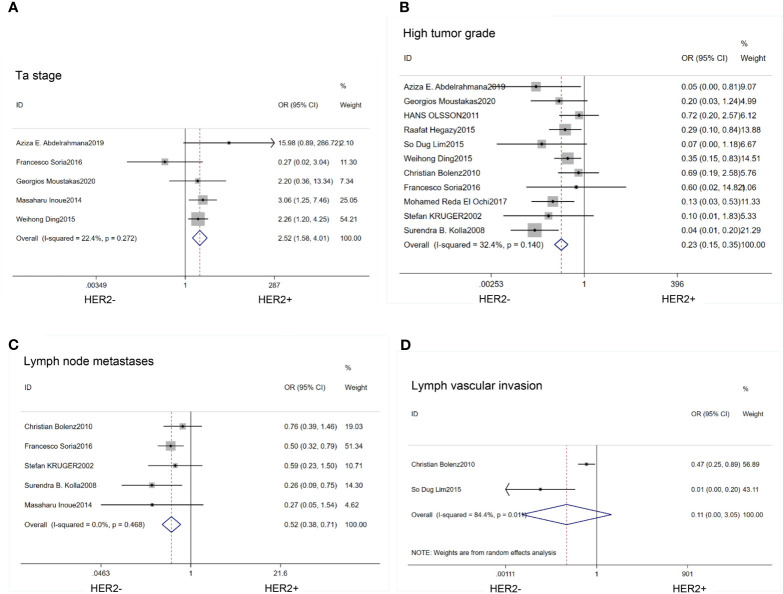
Forest plots describing the correction of HER2 protein expression with stage **(A)**, grade **(B)**, lymph node metastases **(C)** and lymph vascular invasion **(D)**. OR, odds ratio; CI, confidence interval.

We found that HER2 positive rate was high in both carcinoma *in situ* (CIS)(OR=0.62; 95% CI:0.42–0.92; p=0.02) (14, 17, 18, 20, 21, 26, 27) (**Figure 2B**) and multifocal tumors (OR=0.45; 95% CI:0.30–0.68; p<0.01) (14, 17, 18, 21, 27) (**Figure 2C**). HER2 expression was also associated with large tumor size(>3cm) (OR=0.40; 95% CI:0.26–0.63; p<0.01) (14, 17, 20, 23, 27) (**Figure 2D**). In the HER2 positive tumors, the proportion of Ta stage was significantly lower than that in the negative tumors (OR=2.52;95%CI:1,58–4.01; p<0.01) (14, 21, 24, 25, 27) (**Figure 3A**). Furthermore, HER2 expression was linked with high tumor grade (OR=0.23; 95% CI:0.15–0.35; p<0.01) (14–18, 20, 23–27) (**Figure 3B**) and lymph node metastasis (OR=0.52; 95% CI:0.38–0.71; p<0.01) (15, 16, 21, 25, 26) (**Figure 3C**). But the expression level of HER2 protein was not associated with lymph vascular invasion (OR=0.11; 95% CI:0.00–3.05; p=0.19) (17, 26) (**Figure 3D**).

### Comparison of Prognosis

**Figure 4:**
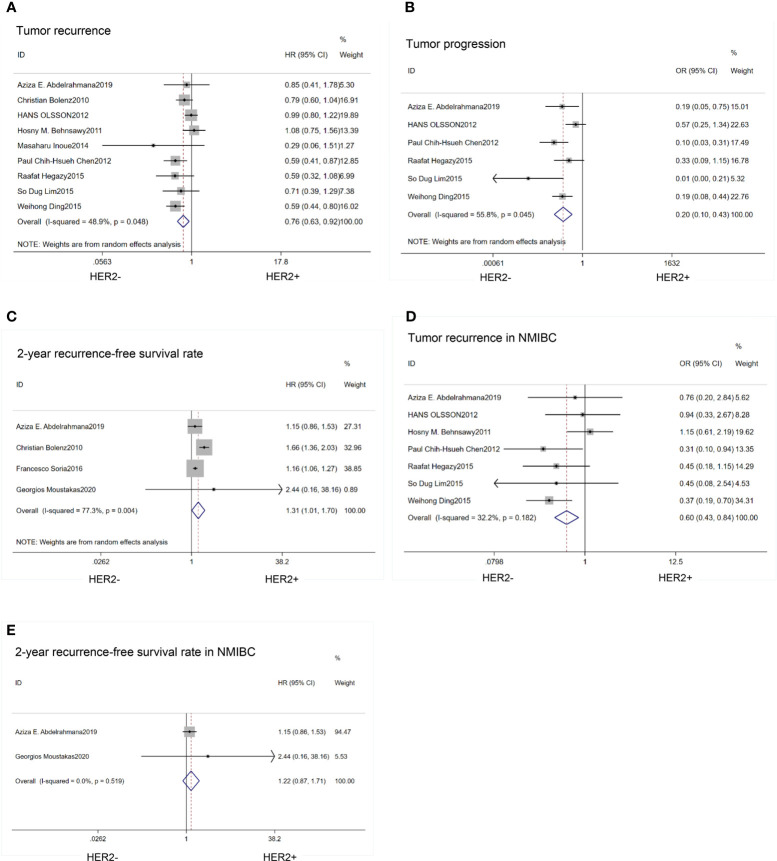
Forest plots describing the correction of HER2 protein expression with recurrence **(A)**, progression **(B)**, 2-year RFS **(C)**, recurrence in NMIBC **(D)**, 2-year RFS in NMIBC **(E)**. RFS, recurrence-free survival; NMIBC, non-muscle invasive bladder cancer. OR, odds ratio; HR, hazard ratio; CI, confidence interval.

Patients with high expression of HER2 protein had a greater risk of tumor recurrence (HR=0.76; 95% CI:0.63–0.92; p<0.01) (14, 17–19, 21–23, 26, 27) (**Figure 4A**) and progression(HR=0.31; 95% CI:0.18–0.54;p<0.01) (14, 17–19, 21–23, 27) (**Figure 4B**) than those with low expression of HER2. HER2 expression was associated with a low 2-year recurrence-free survival (RFS) rate (HR=1.31; 95% CI:1.01–1.70; p=0.04) (24–27) (**Figure 4C**). Progression, recurrence and survival conditions have different impact between MIBC and NMIBC cohorts, so we did subgroup analysis in NMIBC patients. The results were as follows: recurrence (HR=0.77; 95% CI:0.61–0.97; p=0.02) (14, 17, 18, 21–23, 27) (**Figure 4D**); 2-year recurrence-free survival (RFS) (HR=1.22;95%CI:0.87–1.71; p=0.25) (24, 27) (**Figure 4E**).

### TCGA Database Analysis Results

**Figure 5:**
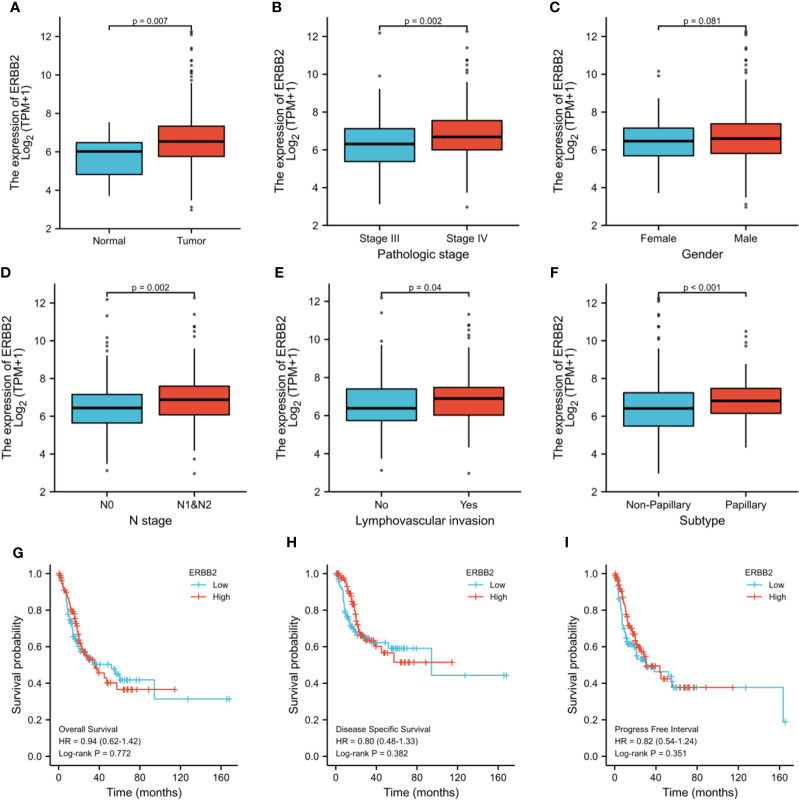
TCGA analysis of differences about HER2 gene expression between cancer and normal tissues **(A)**, stage III and IV **(B)**, male and female **(C)**, N0 and N1&2 tumor **(D)**, LVI and no-LVI **(E)** and subtypes **(F)**. Survival conditions were also compared: overall survival **(G)**, disease specific survival **(H)** and progress free interval **(I)**. ERBB2: HER2, Human Epidermal Growth Factor Receptor 2; LVI, lymph vascular invasion; HR, hazard ratio.

HER2 gene expression in BCa tissues was significantly higher than that in normal tissues (P=0.007) (**Figure 5A**). HER2 expression was significantly elevated in patients with high pathologic stage (P=0.002) (**Figure 5B**). HER2 expression in patients with Bca was independent of gender, which was consistent with our results (P=0.081) (**Figure 5C**). Over-expression of HER2 gene also correlated with lymph node metastases, lymph vascular invasion, and tumor subtype (P<0.05) (**Figure 5D–F**). There were no significant differences in overall survival, disease-specific survival, and progress-free interval between different levels of HRE2 gene amplification groups(P>0.05) (**Figure 5G–I**).

### Publication Bias

**Figure 6:**
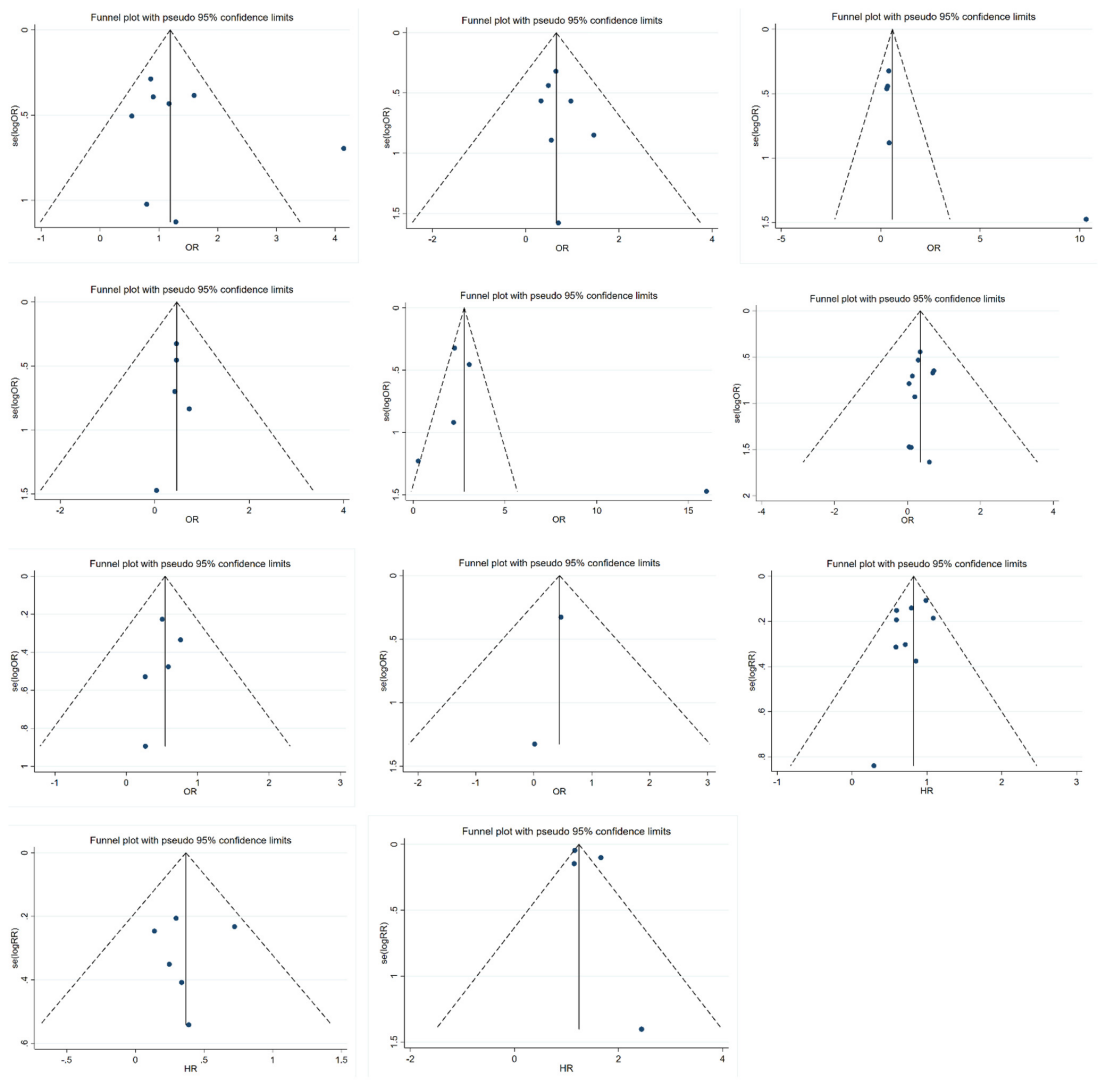
Funnel plots of meta analysis.

Funnel plots were used to detect publication bias, as shown in **Figure 6**. Evidence showed that funnel plots for each group were symmetrical, with no significant risk of bias. This result suggested that the results of this meta-analysis were reliable.

## Discussion

Our study focused on the relationship of HER2 expression in bladder cancer between oncological characteristics and patient prognosis. We included 14 articles, 1398 patients for our study. We also used the TCGA database for analysis. Combining our meta-analysis and TCGA database exploration, we came to a number of conclusions.

First, based on our findings, we learned that HER2 expression was significantly higher in bladder cancer than in normal tissue at both the transcriptional and translational levels. Many years ago, Eissa et al. found significant overexpression of HER2 in the malignant bladder cancer group compared to the benign and normal groups by enzyme immunoassay (13). In 2019, Sanguedolce et al. also declared that HER2 expression was significantly elevated in bladder cancer and this phenomenon was associated with tumor development (30). Thus, it can be seen that HER2 expression is relavent for bladder cancer.

We could not find any relationship between HER2 expression and gender in BCa patients, either by genetic testing or IHC. However, differently, HER2 overexpression was more commonly observed in male gastric cancer patients of Japan (31). Fan et al. came to the same conclusion in a related survey of the Chinese population (32). Thus, the relationship between HER2 in pan-cancer and gender is unclear and needs to be further validated by large-scale clinical studies.

Our meta-analysis displayed that HER2 protein tended to be highly expressed in CIS and multifocal tumors. Similar conclusions have been drawn from previous studies (14, 26). CIS is a flat, noninvasive urothelial carcinoma with a high probability of progression. And CIS is usually multifocal, the incidence of muscle infiltration in CIS tends to be significantly higher than in Ta and T1 stage bladder cancer (33, 34). Also, multifocal CIS has proven to be associated with a high risk of recurrence in BCa (35). We concluded that overexpression of HER2 in BCa often appeared in CIS and multifocal tumors which predicted a high risk of tumor recurrence.

In our meta-analysis study, HER2 protein expression was clearly related to tumor size, grade, and stage of BCa. Also, by analyzing the BCa data from TCGA, we found that HER2 gene expression was associated with tumor stage. The details of the relevant mechanism need to be confirmed in further study.

We found that high expression of both HER2 gene and protein was related to lymph node metastasis in BCa. A previous study has indicated that the probability of HER2 positivity in lymph node metastases was significantly higher than in the primary site of BCa, too (36). Interestingly, this also seemed to be true for other kinds of tumors. Lu et al. claimed that HER2 may serve as a potential biomarker for lymph node metastasis in colorectal cancer (37). In examining the relationship between HER2 and lymphovascular invasion, we found inconsistent results between gene amplification and protein levels. The enhancement of HER2 gene expression seemed to be connected with lymphovascular invasion.

We also found that HER2 protein overexpression was highly correlated with tumor recurrence, progression, and RFS in patients with BCa. A recent study has indicated that HER2 expression in bladder cancer cells was associated with tumor recurrence. In this published literature, Kim *et al.* discussed that there was a correlation between HER2 and immune checkpoint proteins in BCa (38). Similar findings have also been reported over the last few years. Nedjadi et al. revealed that HER2 expression was associated with disease aggressiveness (39). The results of subgroup analysis were consistent. We found that HER2 expression did not affect RFS in NMIBC. TCGA data analysis indicated that HER2 gene was not connected with overall survival, disease-specific survival and progress-free interval in BCa patients.

Due to the conflicting results, variances in HER2 expression among BCa patients remain unclear. After consulting relevant literatures, we speculated that the differential expression of HER2 among people might be related to a wide variety of molecules such as androgen receptor (AR). The AR signaling pathway has been proven to promote tumor development and progression in BCa (40). Zheng et al. claimed that AR activation upregulated the expression of HER2 in bladder cancer cells (41). It has been proved that inhibiting AR pathway can successfully control the occurrence and development of bladder cancer, and can be synergistic with the cisplatin chemotherapy regimen (42). There were other molecules that have been shown to be associated with HER2. For example, the research results of Memon et al. suggested that the final outcome of patients with high HER2 gene expression in BCa depended on the expression of HER3 and HER4 (43).

Although HER2-associated molecular immunotherapy for BCa has not been put to clinical use, the idea has been proposed for a long time and is still emerging (16, 44). Nagasawa et al. found that TAK-165 (a potent inhibitor of HER2) significantly inhibited the growth of bladder cancer cell. It may be a hopeful agent for BCa (45). Tsai et al. constructed a HER2-targeted, envelope-modified retroviral vector which carried the interleukin (IL)-12 gene for the treatment of BCa in mice (46). A prior study demonstrated that epidermal growth factor receptor (EGFR) TKI (tyrosine kinase inhibitors) blocked both radiation-activated EGFR and HER2 signaling and inhibited the growth of BCa cells *in vitro* (47). T-DM1, a drug consisting of the HER2 antibody trastuzumab in combination with a cytotoxic agent, has been indicated to be superior to trastuzumab alone in breast cancer by Hayashi et al. (48). A recent study pointed out that the conjugates of epidermal growth factor and anthrax toxin could be a new approach against BCa in dogs (49). Indoleamine 2, 3-dioxygenase and programmed death ligand-1 have been proved to be associated with HER2 protein. All of these three molecules can be used as directions for immunotherapy in BCa (38).

There are many other studies focusing on HER2-mediated mechanisms of cancer which open the door to new drug development. Mika et al. found the SORLA-dependent molecular pathway in HER2-driven breast cancer cells (50). Yoshihisa et al. suggested that syn-miR-143 down-regulated the expression of HER2 through silencing DEAD/H-box RNA helicase 6 (DDX6) in HER2-positive gastric cancer cells (51). Thus, it seems that HER2-related tumor-targeting research is still very promising. The mechanism of HER2 in bladder cancer still needs to be further explored.

There are several limitations in our study. First, we only searched PubMed database, the number of included literatures is not big enough. Second, the search was restricted to English-language articles, we may have missed some documents written in other languages. Finally, in the meta-analysis literature we included, HER2 expression was measured by the IHC method only. It has been suggested that the results of IHC and fluorescence *in situ* hybridization (FISH) were not completely consistent when detecting HER2 expression in bladder cancer (52, 53). A combination of FISH assays is needed for further research in the future.

## Conclusion

HER2 showed enhanced expression level in bladder cancer in comparison with normal samples and its overexpression was tightly associated with CIS, multifocal tumor, large tumor size, high tumor stage and grade, lymph node metastasis, progression, recurrence, and papillary tumor, indicating it had the potential to become an ideal target for bladder cancer therapy.

More thorough mechanistic research should be carried out to investigate the function of HER2 in bladder cancer.

## Data Availability Statement

The original contributions presented in the study are included in the article/supplementary material. Further inquiries can be directed to the corresponding authors.

## Author Contributions

KG, YG, and KL conceived the idea of the study, did the literature search and selected the studies. KG and YG extracted the relevant information. BX and WQ have carefully revised the manuscript and refined the English writing. All authors contributed to the article and approved the submitted version.

## Funding

The work was supported by the National Natural Science Foundation of China (No. 81772734, 81802935, 81872089).

## Conflict of Interest

The authors declare that the research was conducted in the absence of any commercial or financial relationships that could be construed as a potential conflict of interest.

## Publisher’s Note

All claims expressed in this article are solely those of the authors and do not necessarily represent those of their affiliated organizations, or those of the publisher, the editors and the reviewers. Any product that may be evaluated in this article, or claim that may be made by its manufacturer, is not guaranteed or endorsed by the publisher.

## Abbreviations

BCa, bladder cancer; HER2, Human Epidermal Growth Factor Receptor 2; OR, odds ratio; HR: hazard ratio; CI: confidence interval; TCGA, The cancer genome atlas; RFS, recurrence-free survival; NMIBC, non-muscle invasive bladder cancer; MIBC, muscle invasive bladder cancer; PRISMA: Preferred Reporting Items for Systematic Reviews and Meta-Analysis; PICO: population, intervention, comparison, results; IHC, immunohistochemistry; ASCO, American Society of Clinical Oncology; CAP, American College of Pathology; NOS, Newcastle - Ottawa Quality Assessment Scale; CIS, Carcinoma *in situ*; AR, androgen receptor; EGFR, epidermal growth factor receptor; TKI, tyrosine kinase inhibitors; DDX6, DEAD/H-box RNA helicase 6; FISH, fluorescence *in situ* hybridization; FISH, fluorescence *in situ* hybridization.
